# Paleomagnetic and paleoenvironmental implications of magnetofossil occurrences in late Miocene marine sediments from the Guadalquivir Basin, SW Spain

**DOI:** 10.3389/fmicb.2014.00071

**Published:** 2014-03-04

**Authors:** Juan C. Larrasoaña, Qingsong Liu, Pengxiang Hu, Andrew P. Roberts, Pilar Mata, Jorge Civis, Francisco J. Sierro, José N. Pérez-Asensio

**Affiliations:** ^1^Instituto Geológico y Minero de EspañaMadrid, Spain; ^2^Institute of Earth Sciences Jaume Almera, CSICBarcelona, Spain; ^3^State Key Laboratory of Lithospheric Evolution, Institute of Geology and Geophysics, Chinese Academy of SciencesBeijing, China; ^4^Research School of Earth Sciences, The Australian National UniversityCanberra, Australia; ^5^Departamento de Geologïa, Universidad de SalamancaSalamanca, Spain; ^6^Department of Earth Sciences, University of GenevaGeneva, Switzerland

**Keywords:** Guadalquivir Basin, late Miocene, marine sediments, rock magnetism, magnetotactic bacteria, Messinian salinity crisis

## Abstract

Although recent studies have revealed more widespread occurrences of magnetofossils in pre-Quaternary sediments than have been previously reported, their significance for paleomagnetic and paleoenvironmental studies is not fully understood. We present a paleo- and rock-magnetic study of late Miocene marine sediments recovered from the Guadalquivir Basin (SW Spain). Well-defined paleomagnetic directions provide a robust magnetostratigraphic chronology for the two studied sediment cores. Rock magnetic results indicate the dominance of intact magnetosome chains throughout the studied sediments. These results provide a link between the highest-quality paleomagnetic directions and higher magnetofossil abundances. We interpret that bacterial magnetite formed in the surface sediment mixed layer and that these magnetic particles gave rise to a paleomagnetic signal in the same way as detrital grains. They, therefore, carry a magnetization that is essentially identical to a post-depositional remanent magnetization, which we term a bio-depositional remanent magnetization. Some studied polarity reversals record paleomagnetic directions with an apparent 60–70 kyr recording delay. Magnetofossils in these cases are interpreted to carry a biogeochemical remanent magnetization that is locked in at greater depth in the sediment column. A sharp decrease in magnetofossil abundance toward the middle of the studied boreholes coincides broadly with a major rise in sediment accumulation rates near the onset of the Messinian salinity crisis (MSC), an event caused by interruption of the connection between the Mediterranean Sea and the Atlantic Ocean. This correlation appears to have resulted from dilution of magnetofossils by enhanced terrigenous inputs that were driven, in turn, by sedimentary changes triggered in the basin at the onset of the MSC. Our results highlight the importance of magnetofossils as carriers of high-quality paleomagnetic and paleoenvironmental signals even in dominantly terrigenous sediments.

## INTRODUCTION

Magnetosomes are submicron crystals of magnetite (Fe_3_O_4_) or greigite (Fe_3_S_4_) that grow intracellularly, forming chains, within magnetotactic bacteria (MTB) to assist them in navigation within aquatic environments (Blakemore, 1975; Bazylinski and Frankel, 2004; Faivre and Schüler, 2008; Kopp and Kirschvink, 2008). Aside from having important applications in microbiology and biotechnology (see Faivre and Schüler, 2008), magnetosomes (or magnetofossils when found in the sedimentary record) are important in Earth science because they have ideal sizes (single domain, SD) for recording stable paleomagnetic signals. In addition, variations in magnetofossil abundances in sediments and sedimentary rocks have been interpreted to provide information concerning the response of MTB communities to changing paleoenvironmental conditions (Tarduno and Wilkison, 1996; Lean and McCave, 1998; Tarduno et al., 1998; Yamazaki and Kawahata, 1998; Dinarès-Turell et al., 2003; Roberts et al., 2011; Larrasoaña et al., 2012; Lin et al., 2012; Yamazaki and Ikehara, 2012; Yamazaki et al., 2013).

**FIGURE 1:**
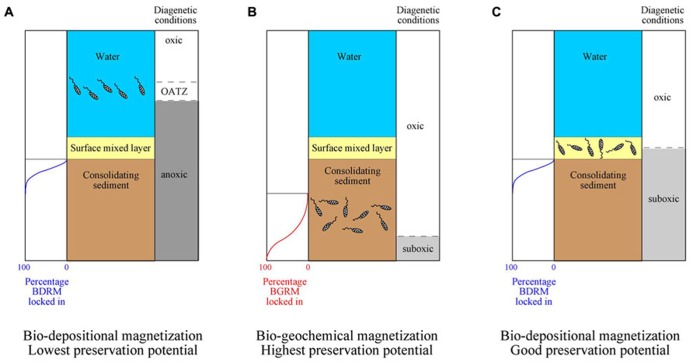
**Schematic diagrams of paleomagnetic and environmental signals recorded by magnetofossils.** **(A)** Magnetofossils produced by magnetotactic bacteria that lived in the water column will behave effectively as detrital particles within the surface mixed layer, and will retain a bio-depositional remanent magnetization (BDRM). Location of the oxic–anoxic transition zone (OATZ) in the water column implies strong anoxic conditions that will result in low preservation potential due to reductive dissolution during subsequent burial. **(B)** Magnetofossils produced by magnetotactic bacteria that lived within consolidating sediments below the surface mixed layer under oxic conditions will give rise to a biogeochemical remanent magnetization (BGRM) with good preservation potential. **(C)** Magnetofossils produced by magnetotactic bacteria that lived within the surface mixed layer will retain a BDRM. Milder, suboxic diagenetic conditions can lead to preservation of magnetofossils throughout thick sedimentary sequences. Based on Roberts et al. (2013). Notice that the alignment of magnetotactic bacteria with the ambient field is much better in water than within sediments (Mao et al., 2014).

Magnetite-producing MTB thrive typically around the oxic–anoxic transition zone (OATZ; Faivre and Schüler, 2008; Kopp and Kirschvink, 2008), although they have also been linked with micro-aerobic environments (Blakemore et al., 1985; Schüler and Baeuerlein, 1998) and even oxic conditions (Yamazaki and Shimono, 2013) decoupled from the occurrence of an OATZ (Roberts et al., 2013). Under reducing diagenetic conditions, the OATZ can occur within the water column or the uppermost centimeters of the sediment column (e.g., the bioturbated surface sedimentary mixed layer). In this case, magnetosome chains that accumulate after bacterial death will behave in the same way as any other detrital grain subjected to pelitization, bioturbation and other processes within the surface mixed layer (Paterson et al., 2013; Roberts et al., 2013; Mao et al., 2014). Magnetofossil chains are expected to adhere onto sediment particles instead of being freely suspended in pore waters, which explains the overall poor alignment of magnetofossil chains (as compared with water) typically reported in MTB-bearing modern sediments (Mao et al., 2014). With ongoing sedimentation and burial, magnetofossils are likely to acquire a magnetization that is essentially identical to a post-depositional remanent magnetization (PDRM) but that, given its origin and distinctive significance, we refer to as a bio-depositional remanent magnetization (BDRM). Magnetofossils in this case should carry a syn-depositional signal that can be used to study short-period geomagnetic field behavior and will provide reliable magnetostratigraphic data. They will also record a paleoenvironmental signal that is contemporaneous with any other sediment constituent (e.g., detrital particles, foraminiferal tests, etc.). Under anoxic conditions, however, preservation of magnetofossils (and detrital magnetic minerals) is unlikely given that reductive dissolution will occur under such conditions (**Figure 1A**; Bazylinski and Frankel, 2004; Faivre and Schüler, 2008; Kopp and Kirschvink, 2008). Such reductive dissolution is responsible for liberation of Fe^2^^+^ that, after its upward flux, is used by MTB to synthesize magnetosomes around the OATZ (Faivre and Schüler, 2008; Kopp and Kirschvink, 2008; Roberts et al., 2011, 2013). Strongly reducing conditions are typical in continental margin sediments, where high organic carbon supply and high accumulation rates favor burial and degradation of organic matter within sediments (Roberts et al., 2013). Magnetofossils might be preserved at discrete intervals due to transient disruption of reducing conditions. This seems to have been the case for magnetofossils that accumulated during the Paleocene-Eocene thermal maximum in the North American Atlantic continental margin ( Kopp et al., 2007; Lippert and Zachos, 2007; Dickens, 2008).

When oxic depositional conditions prevail, it is possible that microaerobic conditions persist throughout the sediment column. In this case, upward diffusion of Fe^2^^+^ liberated by dissolution of the most reactive iron (oxyhydr)oxides [e.g., ferric hydrous oxide, ferrihydrite and lepidicrocite (Poulton et al., 2004)] at greater depths is used by MTB to biomineralize magnetosomes (Roberts et al., 2011, 2013). An extreme case of MTB living in oxic conditions is found in pelagic red clays (Yamazaki and Shimono, 2013). When MTB live within consolidating sediments in these cases, accumulation of magnetosomes after death of the MTB can result in acquisition of a remanent magnetization with an age that will be delayed with respect to that of the host sediment (Tarduno and Wilkison, 1996; Tarduno et al., 1998; Abrajevitch and Kodama, 2009; Roberts et al., 2013; **Figure 1B**). This magnetization is referred to as a biogeochemical remanent magnetization (BGRM; Tarduno and Wilkison, 1996; Tarduno et al., 1998). A BGRM is likely to occur in deep-sea sediments (pelagic carbonates, clays, and oozes), where low organic carbon fluxes, non-zero oxygen contents, and low accumulation rates favor oxidation of most organic matter before it is buried in the sediment, which favors magnetofossil preservation (Roberts et al., 2013). Depths at which BGRMs lock in within pelagic carbonate environments from the equatorial Pacific Ocean have been reported to range from some tens of cm to 4 m, which corresponds to a delay in remanence acquisition between 40 and 420 kyr (Tarduno and Wilkison, 1996). A similar delay of several tens of kyr has been reported by Yamazaki and Shimono (2013) in red clays from the North and South Pacific Ocean. In these cases, magnetofossils are unlikely to provide a depositional signal that can be used to make paleoenvironmental inferences or to study geomagnetic field behavior over short timescales (e.g., secular variation, relative paleointensity, polarity transitions). In contrast, a BGRM is still likely to provide paleomagnetic data that can be used to determine paleomagnetic pole positions and paleolatitude variations, because it will be locked in after initial sediment compaction and will be less affected by inclination shallowing. Magnetostratigraphic data based on BGRMs are likely to be complicated in periods such as the Neogene, when chron duration is often <300 kyr.

When diagenetic conditions are neither strongly reducing, nor strongly oxic, suboxic conditions can be found through thick sedimentary sequences (Roberts et al., 2011). In this case, MTB can live within or below the surface mixed layer, where magnetofossils will give rise to a syn-depositional BDRM or a post-depositional BGRM that is preserved due to subsequent suboxic conditions (**Figure 1C**; Roberts et al., 2013). The fact that most pelagic carbonates and clays provide superb records of geomagnetic polarity changes suggests that such BDRMs are far more common than previously considered in pelagic sediments (Roberts et al., 2013). Suboxic conditions in pelagic settings appear to be linked to fertilization of phytoplankton productivity by eolian dust, which delivers nutrients at concentrations large enough to fuel bacterial metabolism at the seafloor but not in such large amounts to drive reducing diagenetic conditions (Roberts et al., 2011, 2013; Larrasoaña et al., 2012; Yamazaki and Ikehara, 2012).

In recent years, technical improvements aimed at discriminating sources of fine-grained magnetic minerals, such as hysteresis measurements, unmixing of isothermal remanent magnetization (IRM) curves, first-order reversal curve (FORC) diagrams, ferromagnetic resonance (FMR) measurements, high- and low-temperature magnetic measurements, and transmission electron microscope (TEM) observations, enable improved identification of magnetofossils in the sedimentary record (Moskowitz et al., 1993; Egli, 2004; Weiss et al., 2004; Kopp et al., 2006; Liu et al., 2012; Chang et al., 2013; Roberts et al., 2013). Of special relevance is the use of FORC diagrams because they enable conclusive identification of the non-interacting SD properties due to magnetofossils even if they are mixed with other magnetic components. Thus, intact magnetosome chains produce a characteristic “central ridge” on FORC diagrams due to these properties (Yamazaki, 2008; Abrajevitch and Kodama, 2009; Yamazaki, 2009; Egli et al., 2010; Roberts et al., 2011; Larrasoaña et al., 2012; Roberts et al., 2012; Yamazaki and Ikehara, 2012; Channell et al., 2013a,b; Heslop et al., 2013; Paterson et al., 2013; Roberts et al., 2013; Yamazaki et al., 2013). Disrupted magnetosome chains can also be identified because chain disruption increases the magnetic interaction between magnetosome particles and nearby chains (Kind et al., 2011; Li et al., 2012; Roberts et al., 2012, 2013). Improved methods for identifying biogenic magnetite in sediments has enabled rapid expansion of the database of pre-Quaternary magnetofossils in sediments, which until recently were considered rare (Kopp and Kirschvink, 2008). This has boosted renewed interest in the implications of magnetofossils as carriers of paleomagnetic and paleoenvironmental signals (e.g., Roberts et al., 2012, 2013).

Here we present a magnetostratigraphic study of marine sediments from the late Miocene sedimentary fill of the Guadalquivir Basin (GB). These sediments were recovered in two boreholes drilled in the western sector of the basin (Huelva-1 and Montemayor-1). Paleomagnetic data from these sediments have been used to constrain age models for the boreholes (Larrasoaña et al., 2008; Jiménez-Moreno et al., 2013), but details of their paleomagnetic behavior and remanence carriers have not been published previously. Here we provide a description of these paleomagnetic data, which are combined with new hysteresis, FORC, and high- and low-temperature (low-T) measurements to demonstrate that the magnetic mineral assemblage within the studied sediments is dominated by biogenic magnetite. Biostratigraphic and paleoenvironmental data for the studied sediments enable us to discuss the relevance of magnetofossils as reliable carriers of paleomagnetic and paleoenvironmental signals.

## MATERIALS AND METHODS

**FIGURE 2:**
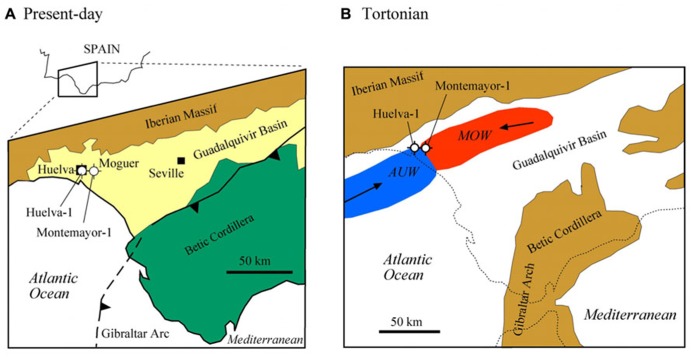
**(A)** Geological sketch map of the lower Guadalquivir Basin, with location of the two studied boreholes (Huelva-1 and Montemayor-1). **(B)** Paleogeographic reconstruction of the Guadalquivir Basin in the late Tortonian (based on Santisteban and Taberner, 1983; Martín et al., 2009), with location of Mediterranean Outflow Water (MOW) and Atlantic Upwelled Water (AUW; after Pérez-Asensio, 2012).

The GB is an ENE-WSW elongated basin that constitutes the foreland of the Betic Cordillera in SW Spain (Sierro et al., 1996; González-Delgado et al., 2004; **Figure 2A**). The GB is limited to the north by the Paleozoic and Mesozoic rocks of the Iberian Massif, and to the South by the Mesozoic and Cenozoic rocks of the Betic Cordillera. During the late Miocene, the GB developed in response to the stacking of tectonic units in the external Betics and the resulting flexural subsidence of the Iberian Massif (Sierro et al., 1996; García-Castellanos et al., 2002; González-Delgado et al., 2004). During the Tortonian stage, the GB constituted the Atlantic side of the Betic Corridors, which, together with the Rifian Corridors in Morocco, enabled connection between the Mediterranean Sea and the Atlantic Ocean (**Figure 2B**). In the lower GB, warm and salty Mediterranean Outflow Waters (MOW) met the cooler and fresher Atlantic Upwelled Waters (AUW; Pérez-Asensio et al., 2012a). Progressive closure of the Betic Corridors led to the GB becoming a wide embayment open to the Atlantic Ocean (Sierro et al., 1996; Martín et al., 2009) and, eventually, to interruption of the connection between the Atlantic Ocean and the Mediterranean Sea during the Messinian salinity crisis (MSC; Krijgsman et al., 1999; Manzi et al., 2013). The sedimentary fill of the GB is made up of a lower marine sequence (late Tortonian–early Pliocene) and an upper continental sequence (late Pliocene to Recent; Sierro et al., 1996; González-Delgado et al., 2004; Salvany et al., 2011).

**FIGURE 3:**
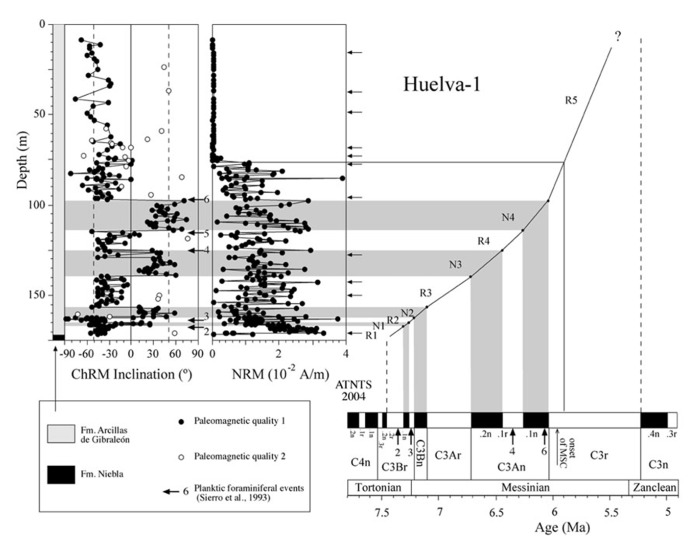
**Magnetostratigraphic and biostratigraphic results from the Huelva-1 borehole, and correlation of polarity intervals with the astronomically tuned Neogene timescale (ATNTS) of Lourens et al. (2004).** Type 1 samples are those with well-defined linear trends that enable accurate calculation of their direction and give rise to optimal polarity determinations. Type 2 samples have poorly developed linear trends and provide doubtful polarity determinations. Arrows indicate the stratigraphic positions of samples used for rock magnetic measurements. Planktic foraminiferal events are: 2, appearance of abundant *G. menardii*, dextral coiling; 3, regular appearance of *G. miotumida*; 4, dextral coiling of *Neogloboquadrina acostaensis*; 5, disappearance of *G. miotumida*; and 6, first abundant occurrence of *G. margaritae*.

**FIGURE 4:**
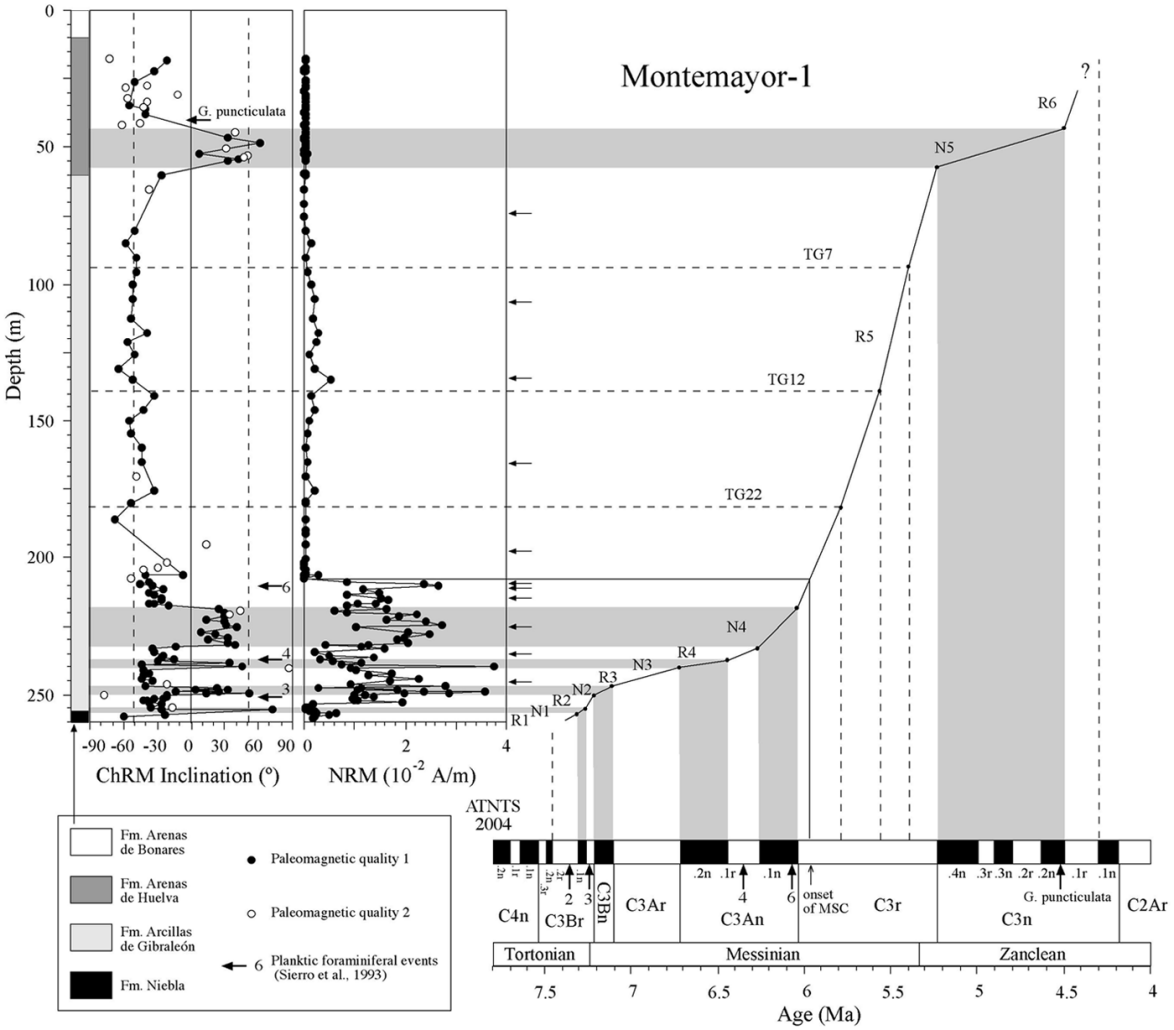
**Magnetostratigraphic and biostratigraphic results from the Montemayor-1 borehole, and correlation of polarity intervals with ATNTS (Lourens et al., 2004).** Additional tie points for the age model are provided by identification of glacial-interglacial stages TG 7, 12, and 22 from oxygen isotope data (Pérez-Asensio et al., 2012a; Jiménez-Moreno et al., 2013). Type 1 and 2 samples are as in **Figure 3**. Arrows indicate the stratigraphic positions of samples used for rock magnetic measurements. Planktic foraminiferal events are as in **Figure 3**.

The Huelva-1 and Montemayor-1 boreholes were drilled by the IGME (Spanish Geological Survey) in the city of Huelva and near the village of Moguer, respectively, in the western sector of the GB (the so-called lower GB; **Figure 2A**). In this part of the basin, the sedimentary sequence reaches its maximum thickness and is not affected by tectonic uplift, so that sediments retain their original horizontal attitude. In the Huelva-1 and Montemayor-1 boreholes, marine sediments were recovered from the three lowermost lithostratigraphic units that constitute the sedimentary fill of the lower GB (Sierro et al., 1996; González-Delgado et al., 2004; **Figures 3** and **4**). The lowermost unit, the Niebla Formation (late Tortonian), is composed of mixed carbonate-siliciclastic coastal deposits that onlap unconformably the Paleozoic–Mesozoic basement (Pendón et al., 2004). The second unit, the Arcillas de Gibraleón Formation (late Tortonian–Messinian), mainly consists of greenish-bluish clays that accumulated in a deep marine trough at the foothills of the Betic Cordillera, and represents the largest volume of sediments in the lower GB (González-Delgado et al., 2004). The uppermost unit, the Arenas de Huelva Formation (early Pliocene), is constituted by sands and silts that accumulated in a shallow marine environment (González-Delgado et al., 2004), and is overlain by transitional sands of the Arenas de Bonares Formation (Mayoral and Pendón, 1987). Continued sedimentation throughout the late Miocene drove the WSW-directed migration of maximum sediment thicknesses along the longitudinal axis of the basin (Sierro et al., 1996). The Huelva-1 borehole encompasses the Niebla Formation (4 m) and most (172 m) of the Arcillas de Gibraleón Formation. The Montemayor-1 borehole includes the Niebla Formation (0.5 m), the Arcillas de Gibraleón Formation (198 m), the Arenas de Huelva (42 m), and the lowermost 14.5 m of the Arenas de Bonares Formation. Both boreholes reached the basement of the basin and include Quaternary deposits in their uppermost meters.

Paleomagnetic samples were taken from the cores parallel to the bedding plane using an electric drill. Sampling resolution ranges between 10 and 100 cm and excluded the uppermost 10–20 m of each core, where the unconsolidated nature of the sediments prevented this type of sampling. The natural remanent magnetization (NRM) was measured using two cryogenic magnetometers (GM400 and 2-G Enterprises) and was demagnetized using a TSD-1 thermal demagnetizer at the Paleomagnetic Laboratory of the Institute of Earth Sciences Jaume Almera (CCiTUB-CSIC), Barcelona. Biostratigraphic results are based on identification of a series of planktic foraminiferal (PF) events, whose determination is based on quantitative and qualitative changes of *globorotaliid* and *neogloboquadrinid* species (Sierro et al., 1993, 1996; González-Delgado et al., 2004).

In order to characterize the magnetic mineralogy of the studied samples, variations in magnetic susceptibility from room temperature to 700°C (χ–T curves) were measured in an argon atmosphere using a Kappabridge KLY-3 magnetic susceptibility meter equipped with a CS-3 furnace. Magnetic hysteresis and FORC measurements were conducted on selected samples to discriminate magnetic mineralogy, domain state, and magnetic interactions among magnetic particles (Day et al., 1977; Roberts et al., 2000). Hysteresis and FORC measurements were made on ~700–800 mg samples using a Princeton Measurements Corporation vibrating sample magnetometer. FORCs were measured following the protocol of Egli et al. (2010) for optimal identification of magnetofossils. We used averaging times of around 0.3–0.5 s and 1 s depending on the magnetization of the sample. FORC diagrams were produced using the VARIFORC software of Egli (2013), which enables variable smoothing that takes into account variable signal-to-noise ratios in different areas within the diagram. Key parameters for VARIFORC calculations (see Egli, 2013 for details) were: *s*_c,0_ = 9, *s*_c,1_ = 9, *s*_b,0_ = 9, *s*_b,1_ = 9, and *λ* = 0.03. Statistical significance levels were calculated for the FORC distributions, and confidence intervals were calculated for profiles through parts of the FORC distributions, following Heslop and Roberts (2012). Low-T measurements of a *M*_r_ applied at 20 K and warmed to room temperature were made with a Quantum Designs Magnetic Property Measurement System (MPMS). These measurements were made after cooling the samples to 20 K both in the presence field cooled (FC) and absence zero-field cooled (ZFC) of a 2.5 T magnetic field. Differences between FC and ZFC low-T measurements, expressed as the normalized difference between the magnetization above and below the Verwey transition for each curve (*δ*_FC_/*δ*_ZFC_), were used to identify intact magnetosome chains in the studied samples and their possible surficial oxidation (Moskowitz et al., 1993). All rock magnetic experiments were performed at the Institute of Geology and Geophysics in Beijing, China.

A representative set of carbon-coated sediment chip fragments was studied using a scanning electron microscope (SEM) to determine the relative amount and microtextures of pyrite, and hence to obtain insights into the presence and strength of reducing diagenetic conditions in the Gibraleón Formation marls. This was done using a Jeol JSM6400, operated at 20 kV, at the National Centre for Electron Microscopy in Madrid. Chemical compositions of sediment constituents were determined using energy dispersive spectroscopy (EDS).

## RESULTS

### MAGNETOBIOSTRATIGRAPHY

**FIGURE 5:**
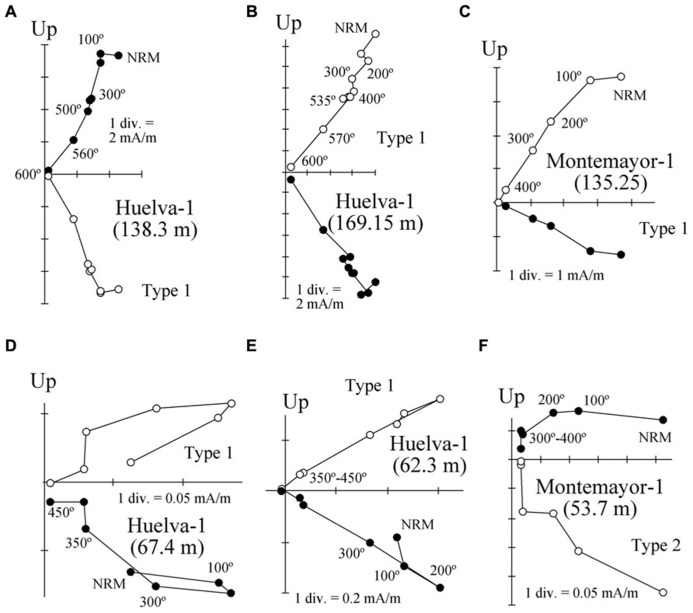
**Representative orthogonal demagnetization diagrams for samples from the Huelva-1 and Montemayor-1 boreholes.** Samples are azimuthally unoriented, so declinations are meaningless. The stratigraphic positions for each sample are indicated in parentheses. **(A–C)** Samples with high NRM intensities. **(D–F)** Samples with weak NRMs.

Thermal demagnetization results indicate the presence of two paleomagnetic components. A low-T component unblocks typically below 200°C and has shallow inclinations. This component is interpreted as an unstable component acquired during drilling, and will not be discussed further. A characteristic remanent magnetization (ChRM) directed toward the origin of the orthogonal vector component plot with both positive and negative directions is identified above 200°C and up to 600°C, which suggests that magnetite is the main carrier of the NRM (**Figure 5**). Stable ChRM directions with unblocking temperatures up to 600°C are typically associated with larger NRM intensities (**Figures 5A,B**). When the NRM is weaker, the ChRM has maximum unblocking temperatures of <450°C but still with a well-defined linear trend directed to the origin of the demagnetization plots (**Figures 5C–E**). Regardless, samples with reliable ChRM directions are labeled as Type 1. Type 2 samples have less clearly identified ChRM directions (e.g., **Figure 5F**), and are not considered further. Type 1 ChRM directions have both positive and negative inclinations regardless of NRM intensity, which suggests that the ChRM provides a reliable record of geomagnetic polarity reversals. The mean of the positive (normal polarity) ChRM directions in the Huelva-1 core is 33.6° ± 17.5°, whereas the mean of the negative (reversed polarity) ChRM directions is -39.4° ± 14.5° (the error given is the standard deviation because the azimuth of the boreholes is unknown and *α*_95_ cannot be calculated). For the Montemayor-1 core, these mean values are 30.6° ± 15.8° and -37.9° ± 12.9°, respectively. The mean values are significantly shallower than the expected inclination for a geocentric axial dipole field at the studied site latitude during the late Miocene (over 50°). The azimuth of the boreholes is unknown, therefore the magnetic polarity stratigraphy is based on the ChRM inclination. Eleven polarity intervals are documented (labeled N1–N5 and R1–R6 for normal and reversed polarity intervals, respectively). The Huelva-1 core includes magnetozones R1–R5, whereas the Montemayor-1 core includes magnetozones R1–R6. The polarity record has a largely square-wave shape that attests to its quality. The exceptions are magnetozones N3 and R4 in the Huelva-1 borehole (**Figure 3**), where reversed and normal polarity directions are recorded down to 2.7 and 4.6 m below their upper boundaries, respectively. A sharp decrease in NRM intensity occurs at around 208 m and 76 m in the Montemayor-1 and Huelva-1 boreholes, respectively (**Figures 3** and **4**).

Planktic foraminiferal event 2 (appearance of abundant *Globorotalia menardii*, dextral coiling) is identified in the uppermost part of magnetozone R1 in the Huelva-1 borehole (**Figure 3**). PF3 (regular appearance of *G. miotumida*, a marker for the Tortonian/Messinian boundary) and PF4 (dextral coiling of *Neogloboquadrina acostaensis*) are identified within magnetozone R2 and at the top of magnetozone N3, respectively, in both boreholes (**Figures 3** and **4**). PF 5 (disappearance of *G. miotumida*) is only identified in the Huelva-1 borehole, in the uppermost part of magnetozone R4. PF6 (first abundant occurrence of *G. margaritae*) has been identified in both boreholes near the magnetozone N4/R5 boundary. The appearance of *G. puncticulata* is found in the lower part of magnetozone R6 in the Montemayor-1 core.

The magnetozone pattern and the position of PF events enable straightforward correlation of the studied cores to the astronomically tuned geomagnetic polarity timescale (ATNTS2004) of Lourens et al. (2004; **Figures 3** and **4**). PF2, PF3, and PF4 are located within chrons C3Br.2r, C3Br.1r and the lower part of C3An.1r, respectively (Lourens et al., 2004). Thus, magnetozone R1 must correlate with chrons C3Br.2r, R2 with C3Br.1r and R4 with C3An.1r. This correlation implies that the long magnetozone R5 correlates with C3r, which is consistent with the long duration of this chron and with the presence of PF6 near the boundary with chron C3An.1n. The appearance of *G. puncticulata* is dated at 4.52 Ma near the top of chron C3n.2n (Lourens et al., 2004). Keeping in mind the litoral facies of the Arenas de Huelva Formation and the possible lack of continuity in such facies, this suggests that magnetozone N5 represents an amalgamation of the lower part of chron C3n due to discontinuous sedimentation (Jiménez-Moreno et al., 2013). In this case, magnetozone R6 probably correlates with chron C3n.1r. The magnetobiostratigraphic results presented here indicate that the Huelva-1 borehole is continuous and spans from the latest Tortonian (chron C3Br.2r, ca. 7.4 Ma) to the latest Messinian (uppermost part of chron C3r, ca 5.4 Ma). The Montemayor-1 borehole spans from the latest Tortonian (chron C3Br.2r, ca 7.4 Ma) to the lower Pliocene (Zanclean, chron C3r/C2Ar boundary, ca 4.3–4.2 Ma), which provides a continuous late Miocene record. The only stratigraphic gap appears to be at the base of the Huelva Formation, which marks a sedimentary break that is attributed to a combination of sea level changes (González-Delgado et al., 2004) and tectonic processes (Salvany et al., 2011). Oxygen isotopic data for benthic and planktic foraminifera from the Montemayor-1 borehole enable identification of three distinctive glacial-interglacial stages (TG7, 12 and 22) within chron C3r, which enable refinement of the age model within this long chron (Pérez-Asensio et al., 2012a; Jiménez-Moreno et al., 2013). The age models thereby established for the Huelva-1 and Montemayor-1 boreholes enable calculation of linear sediment accumulation rates (SARs), which have similar trends at both boreholes (**Figures 3** and **4**). SARs were < 5 cm/kyr until around 6.4 Ma with the exception of a transient increase (up to 8–14 cm/kyr) near the Tortonian/Messinian boundary. After 6.4 Ma, SARs progressively increased and underwent a major rise (exceeding 15 cm/kyr) at around 6 Ma, broadly coincident with the onset of the MSC.

### ROCK MAGNETISM

**FIGURE 6:**
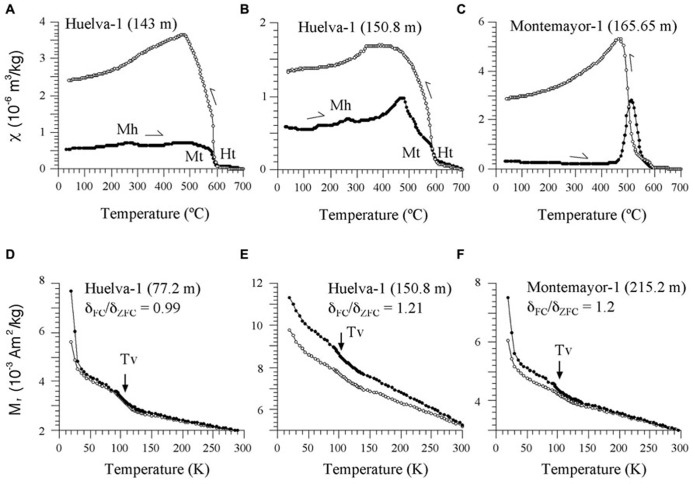
**(A–C)** Representative χ–T curves for samples from the Huelva-1 and Montemayor-1 boreholes. Mt, magnetite; Ht, hematite; Mh, maghemite. **(D–F)** Representative low-T experiments for samples from the Huelva-1 and Montemayor-1 boreholes, with indication of the Verwey transition (Tv) and *δ*_FC_/*δ*_ZFC_ ratios of Moskowitz et al. (1993). The stratigraphic positions for each sample are indicated in parentheses.

Our rock magnetic study focuses on the clays of the Gibraleón Formation because they represent most of the recovered sequences and, as opposed to the Huelva and Niebla formations, have high NRM intensities and sedimentary facies suitable for hosting biogenic magnetite. The main decay observed in the χ–T heating curves for samples located below 208 m and 76 m in the Montemayor-1 and Huelva-1 boreholes, respectively, which are characterized by high NRMs, is a drop at around 580°C (**Figures 6A,B**). This drop, which is sometimes preceded by a Hopkinson peak (**Figure 6B**), indicates the presence of magnetite. Above 580°C, the magnetic susceptibility signal persists and does not disappear completely until about 680°C (**Figures 6A,B**), which indicates variable contributions from hematite. In many samples, a subtle hump can be observed superimposed on the χ–T curve just below 300°C (**Figures 6A,B**). This can be interpreted as due to the thermally induced breakdown of minor amounts of maghemite (Liu et al., 2012). Cooling curves typically have higher χ values than heating curves below 580°C, which indicates widespread formation of magnetite during heating (**Figures 6A,B**). In samples above 208 m and 76 m in the Montemayor-1 and Huelva-1 boreholes, respectively, which are characterized by lower NRMs, thermally induced formation of magnetite above 450°C completely obscures the χ signal (**Figure 6C**). In these cases, a major increase in χ in the cooling curve below 530°C might suggest that the newly formed magnetite is not stoichiometric.

**FIGURE 7:**
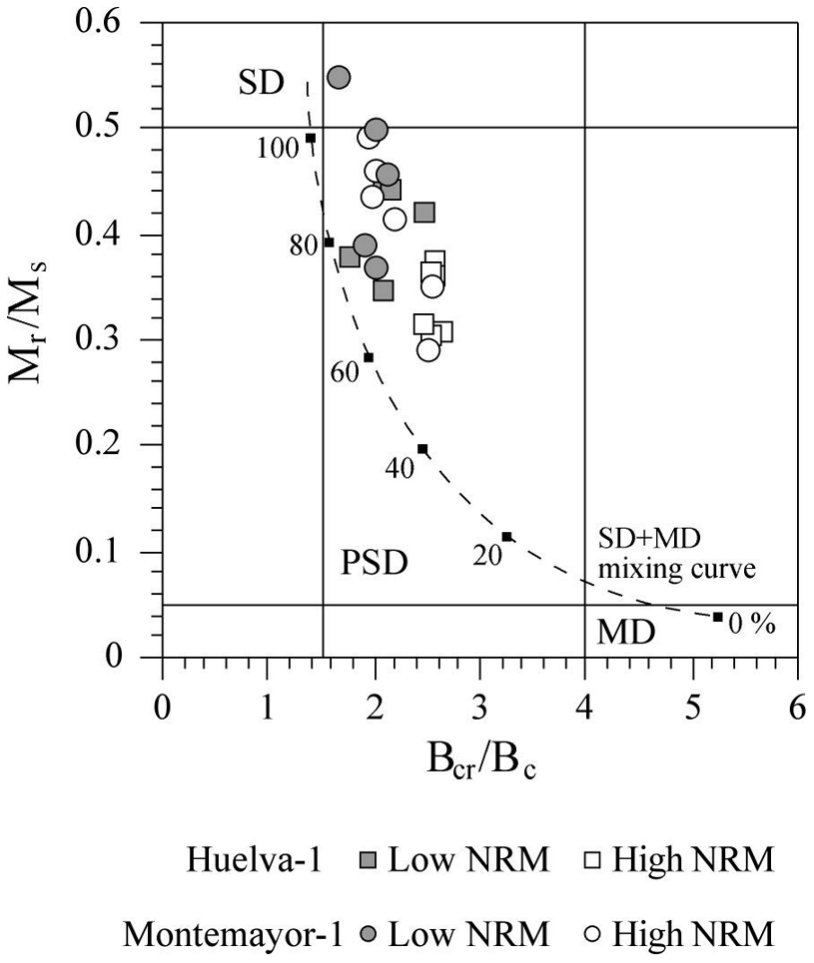
**Magnetic hysteresis results from the Huelva-1 and Montemayor-1 boreholes plotted on a Day diagram (Day et al., 1977).** The line represents a theoretical mixing curve for MD particles at variable percentages (black squares) with uniaxial SD magnetite (Dunlop, 2002). The SD, PSD, and MD labels denote the fields for single-, pseudosingle- and multi-domain particles (Day et al., 1977).

Hysteresis loops for all of the studied samples saturate below 200 mT, which is consistent with the dominance of magnetite. All samples lie between the SD field and the upper left-hand part of the pseudo-single domain (PSD) region of the Day plot (Day et al., 1977; **Figure 7**). Samples lie slightly to the right of the theoretical line that represents mixtures of about 60–100% of equidimensional SD magnetite with multi-domain (MD) magnetite (Dunlop, 2002), which suggests an additional contribution from superparamagnetic (SP) material. The mean M_r_/M_s_ value for Huelva-1 and Montemayor-1 samples is 0.37 and 0.43, respectively, which is close to the theoretical value of 0.5 for uniaxial SD magnetite grains and similar to other sediments where biogenic SD magnetite dominates the magnetic mineral assemblage (Kopp et al., 2007; Lippert and Zachos, 2007; Roberts et al., 2011; Larrasoaña et al., 2012; Roberts et al., 2012, 2013). Samples from the upper parts of the two boreholes, where NRM intensities are distinctively lower, are characterized by larger M_r_/M_s_ values (**Figure 7**), which points to a larger relative contribution of SD magnetite in these sediments.

**FIGURE 8:**
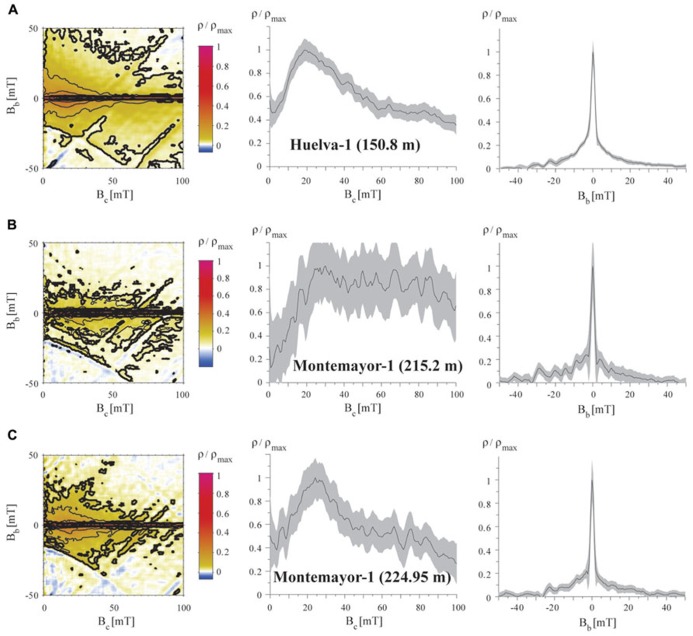
**Representative FORC diagrams for samples from the Gibraleón Formation in the Huelva-1 (A) and Montemayor-1 (B,C) boreholes, plotted with horizontal and vertical profiles through the FORC distributions.** The stratigraphic position for each sample is shown in parentheses. All samples have a central ridge signature typical of intact magnetosome chains (Egli et al., 2010), with variable contributions from other detrital magnetic particles and some magnetic interactions that result from partial collapse of magnetosome chains. VARIFORC parameters used to calculate the FORC distributions (Egli, 2013) are listed in Section 2. Confidence intervals on the profiles were calculated following Heslop and Roberts (2012).

First-order reversal curve distributions of samples from the two studied boreholes are similar in all cases. They are characterized by closed concentric contours about a central peak located between 20 and 30 mT (**Figure 8**). Vertical profiles through the peak of the coercivity distributions have a narrow distribution (e.g., <5 mT) around the dominant central peak, whereas horizontal profiles along *B*_b_ = 0 mT have a skewed Gaussian shape with variable spreading. These features, which are statistically significant at the 0.05 level (see dark contours calculated following Heslop and Roberts (2012) in **Figure 8**), define the “central ridge” signature that is typical of intact magnetosome chains (Egli et al., 2010; Roberts et al., 2011, 2012, 2013). Variable coercivity peaks likely indicate mixtures of the so-called “biogenic soft” and “biogenic hard” magnetosome components, whose coercivities (of about 20 and 40 mT, respectively) likely reflect different magnetosome morphologies (Egli et al., 2010; Yamazaki and Ikehara, 2012; Roberts et al., 2013). In some cases (**Figures 8A,C**), the slight spreading of the outer contours around the peak of the FORC distributions points to some magnetostatic interactions due likely to disruption of a portion of the total number of magnetosome chains (Egli et al., 2010; Kind et al., 2011; Li et al., 2012; Roberts et al., 2012, 2013). The background magnetization observed in the vertical profiles below the narrow central peak suggests an additional contribution of coarser-grained magnetite of probable detrital origin. This detrital component appears to also be responsible for the large spreading observed in the horizontal profile in some samples (**Figure 8B**). The overall weaker magnetization of samples from the upper parts of boreholes Huelva-1 and Montemayor-1 made high-resolution FORC measurements too noisy to enable reliable identification of magnetic components. Nevertheless, hysteresis ratios of weakly magnetic samples are similar to those of samples with a clear central ridge signature in the FORC diagrams, which suggests that magnetofossils also dominate their magnetic assemblages.

Low-T data indicate the presence of the Verwey transition at around 105 K both in the FC and ZFC curves in all samples (**Figures 6D–F**). The FC and ZFC cooled curves typically diverge below the Verwey transition, which gives further support for the occurrence of magnetofossils in the studied samples. *δ*_FC_/*δ*_ZFC_ values range between ~1 and 1.21, which suggests that the magnetosome surfaces are partially oxidized (Moskowitz et al., 1993). This is consistent with the occurrence of maghemite as evidenced by the χ–T curves (**Figures 6A–C**).

**FIGURE 9:**
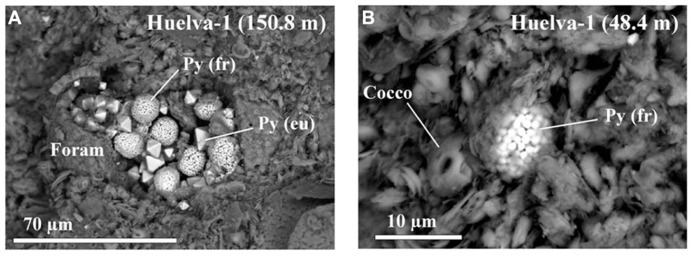
**Representative back-scattered scanning electron microscope images of pyrite microtextures in the Gibraleón Formation clays.** **(A)** Framboidal and euhedral pyrite infilling a calcareous foraminifer shell dispersed within a matrix dominated by clays and quartz. **(B)** Framboidal pyrite located beside a coccolith embedded within the sediment matrix. The stratigraphic positions for each sample are indicated in parentheses. Py (fr), framboidal pyrite; Py (eu), euhedral pyrite crystal; Foram, foraminifer shell; Cocco, coccolith.

Scanning electron microscope observations indicate that the studied sediments contain minor amounts of pyrite that occur typically within foraminiferal tests or near other organic remains such as coccolithophore shells (**Figure 9**). Most pyrite occurs in framboids that are <15 μm in diameter and that consist of small (<1 μm) individual crystals. Framboidal pyrite is often accompanied by euhedral pyrite crystals that are typically <15 μm in size (**Figure 9A**).

## DISCUSSION

### PALEOMAGNETIC IMPLICATIONS OF MAGNETOFOSSIL PRESERVATION

**FIGURE 10:**
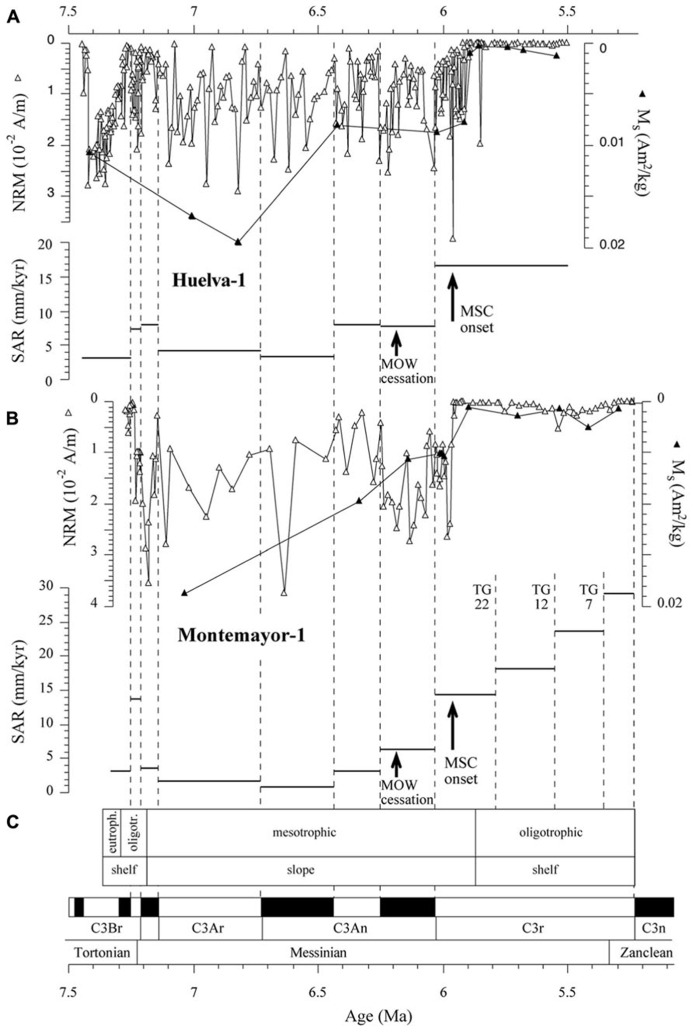
**NRM, M_s_ and sediment accumulation rate (SAR) variations as a function of age for the Huelva-1 (A) and Montemayor-1 (B) boreholes, shown with trophic conditions and depositional context (C) inferred from benthic foraminiferal assemblages in the Montemayor-1 borehole (Pérez-Asensio, 2012; Pérez-Asensio et al., 2012a).** Cessation of flow of the Mediterranean Outflow Water (MOW) along the Betic Corridors (Pérez-Asensio et al., 2012a) and onset of the Messinian salinity crisis (MSC; Manzi et al., 2013) are also shown. Dashed vertical lines mark the tie points used in the age models, including glacial-interglacial (TG) stages.

χ–T and low-T experiments, coupled with hysteresis and FORC results, indicate that the magnetic mineral assemblage of the Gibraleón Formation clays is dominated by fossilized intact magnetosome chains that are in some cases partially oxidized. Significant disruption of magnetofossil chains might have been prevented by adhesion of magnetofossil chains onto the surface of clay particles (e.g., Mao et al., 2014). In the absence of TEM images, an alternative origin for fine-grained magnetite linked to diagenetic oxidation of pyrite might be proposed (e.g., Brothers et al., 1996; Rowan and Roberts, 2006). We discard this possibility because: (1) pyrite in the studied clays is never observed in association with iron oxide overgrowths (**Figure 9**), and (2) this process often occurs during late diagenesis and would result in a prominent paleomagnetic overprint (Brothers et al., 1996; Rowan and Roberts, 2006) rather than in a pristine magnetostratigraphic record (**Figures 3** and **4**). Nevertheless, TEM observations are needed to corroborate the occurrence of magnetofossils in the studied clays and to assist in identifying different magnetosome morphologies. The Gibraleón Formation clays also contain variable amounts of hematite, which is a common constituent of Saharan dust transported into the NE Atlantic Ocean and has been reported in Quaternary sediments off the SW Iberian margin (Channell et al., 2013b). An eolian origin is therefore most likely for the hematite in the studied clays, although its contribution to the NRM is negligible (**Figure 5**). The NRM intensity of the clays is linked to the concentration of magnetofossils, as indicated by the similar NRM and M_s_ variations in both studied boreholes (**Figure 10**). This indicates that the magnetofossil concentration affects the quality of paleomagnetic data by enhancing the NRM. The mean of both normal and reversed polarity directions for the two studied boreholes is 15° to 20° shallower than expected. Inclination shallowing in fine-grained marine sediments has been typically interpreted as due to rolling of magnetic particles as they are deposited on a substrate for a DRM (e.g., King, 1955) or to sediment compaction that will affect a PDRM carried by detrital magnetic particles (Kent, 1973). Given that the magnetic mineral assemblage in the studied sediments is dominated by intact magnetosome chains, which are typically elongated with a length/width ratio that exceeds five (see Faivre and Schüler, 2008), a likely explanation for the observed paleomagnetic inclination shallowing is depositional flattening of magnetosome chains into the bedding plane. Subsequent sediment compaction might have also contributed to the observed inclination flattening, especially if magnetosome chains are adhered onto clay particles (Mao et al., 2014). More detailed studies are necessary to separate the contribution from these processes, which likely operate in concert in clay-rich lithologies that contain intact magnetofossil chains. Methods that enable discrimination between subfabrics of paramagnetic (e.g., low-T AMS) and ferrimagnetic minerals (e.g., AARM) would be of special relevance. Regardless, magnetofossils from the Gibraleón Formation are interpreted to carry a BDRM, which has recorded all polarity chrons regardless of their short durations, especially near the Tortonian/Messinian boundary (e.g., <50 kyr; **Figures 3** and **4**). Possible delayed magnetizations have only been found down to 2.7 and 4.6 m below the uppermost boundaries of chrons C3An.1r and 2n, respectively, in the Huelva-1 borehole (**Figure 3**). Keeping in mind SARs of about 4 and 8 cm/kyr for these chrons, respectively (**Figure 10**), a delay of 68 and 58 kyr, respectively, can be estimated for the lock in time of magnetizations carried by magnetofossils at these depths. Higher-resolution studies are necessary to confirm and better characterize any BGRM in the studied boreholes.

### PALEOENVIRONMENTAL IMPLICATIONS OF MAGNETOFOSSIL PRESERVATION

Benthic foraminiferal assemblages and sedimentary facies indicate that clays of the Gibraleón Formation accumulated in a slope environment, although its lowermost (until about 7.2 Ma) and uppermost (after about 5.85 Ma) parts (**Figure 10**) accumulated in outer continental shelf environments (Pérez-Asensio, 2012; Pérez-Asensio et al., 2012b). Benthic foraminiferal assemblages point to oligotrophic and mesotrophic conditions during deposition of most of this formation, with the exception of one short-lived interval before 7.2 Ma (**Figure 10**; Pérez-Asensio, 2012). Oxygen isotopic data indicate that the lower GB was flushed by both the MOW and the AUW until 6.18 Ma (Pérez-Asensio, 2012; Pérez-Asensio et al., 2012a). After 6.18 Ma, rapid reduction and cessation of flow of the MOW suggests closure of the last marine Betic Corridors (Pérez-Asensio et al., 2012a; **Figure 10**). Later, at about 5.97 Ma, the onset of the MSC led to disconnection of the Mediterranean basin from the Atlantic Ocean (Manzi et al., 2013). Our results indicate that magnetofossils dominate the magnetic mineral assemblage throughout the Gibraleon Formation regardless of changes in depositional setting, nutrient conditions, SARs, and important paleoceanographic events such as cessation of flow of the MOW and onset of the MSC (**Figure 10**). Mild, suboxic diagenetic conditions are required for preservation of magnetofossils through expanded sections of pelagic sediments that record a syn-depositional remanent magnetization (Roberts et al., 2011, 2013). Clays from the Gibraleón Formation are greenish-bluish in colour, which suggests that iron has been reduced and that sulphate reduction might have occurred. It should be noticed, however, that χ–T and low-T experiments indicate that magnetosomes are partially converted into maghemite by surficial oxidation, and it is unlikely that maghemite would have survived reductive dissolution (Smirnov and Tarduno, 2000). SEM observations of pyrite microtextures indicate that reducing diagenetic conditions in clays from the Gibraleón Formation were mild and closely linked with organic-rich microenvironments (e.g., within foraminiferal tests). We interpret that flushing of the bottom waters in the lower GB by either the MOW and/or the AUW, coupled with mostly oligotrophic and mesotrophic conditions, led to mild diagenetic conditions suitable for flourishing of MTB and preservation of their magnetofossils throughout the Gibraleón Formation. Our results expand the settings in which magnetite magnetofossils can dominate the magnetic properties of sediments to include continental margin sedimentary sequences.

**FIGURE 11:**
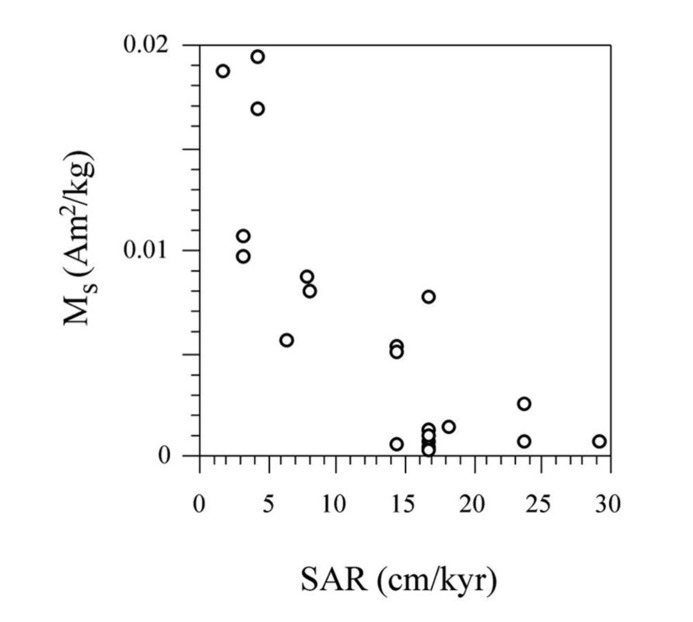
**Illustration of the inverse correlation between M_s_, which is taken as a proxy for the concentration of magnetofossils, and estimated SAR**.

In view of the mild diagenetic conditions that favored preservation of magnetofossils throughout the Gibraleón Formation, and keeping in mind that they largely appear to carry a BDRM that is affected by inclination shallowing, we interpret that MTB lived within the surface mixed layer and, therefore, carry a reliable syn-depositional paleoenvironmental signal. In this case, the main feature needing explanation is the sharp drop observed in magnetofossil abundance at 5.9 Ma and 5.97 Ma in the Huelva-1 and Montemayor-1 boreholes, respectively (**Figure 10**). Pollen data from the Montemayor-1 borehole indicate that glacial/interglacial variability reported at orbital timescales was not significantly modified after the onset of the MSC at 5.97 Ma (Jiménez-Moreno et al., 2013), which suggests that climate variability is not the underlying cause for the observed drop in magnetofossil abundances. In both boreholes, the decreased magnetofossil content predates the change from meso- to oligotrophic conditions and from slope to shelf environments at 5.85 Ma, and postdates cessation of flow of the MOW by more than 200 kyr (**Figure 10**). In the Montemayor-1 borehole, however, the sharp shift in magnetofossil abundance coincides strikingly with the onset of the MSC at 5.97 Ma (Manzi et al., 2013; **Figure 10B**). It should be noted that the age of the drop was established by assuming a linear SAR between the two nearest tie points, namely the chron C3r/C3An boundary and glacial stage TG22 (**Figure 10B**). For the Huelva-1 borehole, the relevant tie points are the chron C3r/C3An boundary and the base of the Huelva Formation, which is located about 20 m higher in the borehole and, according to results from Montemayor-1, is dated near the Miocene/Pliocene boundary (**Figure 4**). Keeping in mind this coarser age constraint, we interpret that the sharp drop in magnetofossil abundances is synchronous at the two boreholes and coincident with the MSC onset. The estimated SARs are also constrained by the positions of tie points used to construct the age models. We therefore associate the drop in magnetofossil abundance to a major rise in SAR at the chron C3r/C3An boundary (6.033 Ma), which follows a steady increase that started at 6.5 Ma (**Figure 10**). This link between magnetofossil abundance and SAR is supported by the overall inverse correlation between M_s_ and SAR (**Figure 11**), and therefore suggests that magnetofossil concentrations are controlled by terrigenous dilution. High-frequency changes in NRM intensity also suggest changes in magnetofossil abundance over much shorter timescales, but the extent and significance of such changes need to be assessed with more detailed rock-magnetic data. The marked rise in SAR appears to be linked to the onset of the MSC at 5.97 Ma (Manzi et al., 2013). We tentatively attribute this increased SAR to a change in sedimentation pattern in the GB during the MSC. Thus, closure of the Betic Corridors at that time might have conditioned delivery of large amounts of sediment that resulted from denudation of the Betic Cordillera, which was previously deposited in the Mediterranean Sea, into the Atlantic Ocean. This, in turn, would have accelerated westward progradation of sedimentary systems along the axis of the GB and the arrival of enhanced terrigenous inputs to the locations of the studied boreholes. Regardless of this interpretation, our results indicate that magnetofossils from the Gibraleón Formation carry paleoenvironmental signals that will help to better constrain the paleoceanographic and sedimentary evolution of the GB during the MSC.

## CONCLUSIONS

Our results indicate that the magnetic mineral assemblage from the late Miocene Gibraleón Formation is dominated by intact magnetofossil chains. We suggest that ventilation of bottom waters in the lower GB, coupled with mostly oligotrophic and mesotrophic conditions, led to sustained suboxic diagenetic conditions suitable for flourishing of MTB and preservation of their magnetofossils throughout the Gibraleón Formation. Our results expand the range of settings in which magnetofossils can dominate the magnetic properties to include expanded continental margin sedimentary sequences, provided that diagenetic conditions remained suboxic and not anoxic. The concentration of magnetofossils also determines the quality of paleomagnetic data, which provides a robust magnetostratigraphic chronology for the studied sedimentary sequence despite recording paleomagnetic inclinations that are 15–20° shallower than expected. This observation is interpreted to indicate that MTB lived within the sedimentary surface mixed layer, so that magnetofossils were affected by sediment compaction upon burial. Our results might also indicate depositional flattening of magnetosome chains due to their large length/width ratio. Regardless, our results suggest that magnetofossils carry a magnetization that is essentially identical to a post-depositional remanent magnetization, which, given its origin and distinctive significance, we refer to as a BDRM. The only exceptions to this syn-depositional pattern of remanence acquisition occur at the tops of chrons C3An.1r and 2n, where paleomagnetic directions appear to be delayed by ~60–70 kyr. Magnetofossils in these cases are interpreted to carry a BGRM. Magnetofossil abundances decrease sharply in the two studied boreholes that coincided, within the limits of the age model, with a major rise in sediment accumulation rate near the onset of the MSC. Sedimentary changes triggered in the GB at the onset of the MSC appear to have caused enhanced terrigenous inputs and dilution of magnetofossil abundances. Our results indicate that magnetofossils can carry high-quality paleomagnetic data and useful paleoenvironmental signals even in dominantly terrigenous sediments.

## AUTHOR CONTRIBUTIONS

Juan C. Larrasoaña, Pilar Mata and Jorge Civis designed the study. Juan C. Larrasoaña, Francisco J. Sierro and Jorge Civis produced magnetobiostratigraphic data. Qingsong Liu, Pengxiang Hu, and Andrew P. Roberts provided and processed rock magnetic data. José N. Pérez-Asensio provided paleoenvironmental data from the Montemayor-1 borehole. Juan C. Larrasoaña led the writing of the paper, with input from all co-authors.

## Conflict of Interest Statement

The authors declare that the research was conducted in the absence of any commercial or financial relationships that could be construed as a potential conflict of interest.
